# 8-Oxoguanine DNA Glycosylases: One Lesion, Three Subfamilies

**DOI:** 10.3390/ijms13066711

**Published:** 2012-06-01

**Authors:** Frédérick Faucher, Sylvie Doublié, Zongchao Jia

**Affiliations:** 1 Department of Biomedical and Molecular Sciences, Queen’s University, 18 Stuart Street, Kingston, K7L 3N6, Canada; 2 Department of Microbiology and Molecular Genetics, University of Vermont, E314A Given Building, 89 Beaumont Avenue, Burlington, VT 05405, USA; E-Mail: Sylvie.Doublie@uvm.edu

**Keywords:** 8-oxoguanine DNA glycosylase, DNA repair, OGG, 8-oxoguanine, base excision repair, crystallography, protein-DNA complex

## Abstract

Amongst the four bases that form DNA, guanine is the most susceptible to oxidation, and its oxidation product, 7,8-dihydro-8-oxoguanine (8-oxoG) is the most prevalent base lesion found in DNA. Fortunately, throughout evolution cells have developed repair mechanisms, such as the 8-oxoguanine DNA glycosylases (OGG), which recognize and excise 8-oxoG from DNA thereby preventing the accumulation of deleterious mutations. OGG are divided into three subfamilies, OGG1, OGG2 and AGOG, which are all involved in the base excision repair (BER) pathway. The published structures of OGG1 and AGOG, as well as the recent availability of OGG2 structures in both apo- and liganded forms, provide an excellent opportunity to compare the structural and functional properties of the three OGG subfamilies. Among the observed differences, the three-dimensional fold varies considerably between OGG1 and OGG2 members, as the latter lack the A-domain involved in 8-oxoG binding. In addition, all three OGG subfamilies bind 8-oxoG in a different manner even though the crucial interaction between the enzyme and the protonated N7 of 8-oxoG is conserved. Finally, the three OGG subfamilies differ with respect to DNA binding properties, helix-hairpin-helix motifs, and specificity for the opposite base.

## 1. Introduction

### 1.1. Guanine Oxidation

DNA contains various moieties that can react with a vast array of chemicals and different types of radiation. Amongst the agents threatening the integrity of DNA, oxidation is particularly common. Reactive oxygen species (ROS) can be generated via exogenous sources as well as from the cell’s own environment and metabolism [1]. Because of its low redox potential [2,3] caused by the unsaturated N7–C8 bond, guanine is prone to oxidation, which generates the most common oxidation product, 7, 8-dihydro-8-oxoguanine (8-oxoG) [4]. 8-oxoG constitutes the most frequent base lesion observed in DNA with an estimated frequency of ~0.3–4 lesions/10^6^ bases [5]. For that reason, the presence of 8-oxoG is commonly used as a cellular biomarker of oxidative stress [6]. Oxidation of guanine is modulated by sequence context. It has been shown both by *ab initio* calculations and by experimentation that the 5′-G of consecutives G’s (*i.e*., GGG, GG) is more prone to oxidation than a lone G in DNA [7,8]. In this context, it is speculated that the GC rich regions outside DNA coding regions may act as “an oxidation pool” to protect genes [9].

### 1.2. Miscoding Properties of 8-oxoG

**Figure 1:**
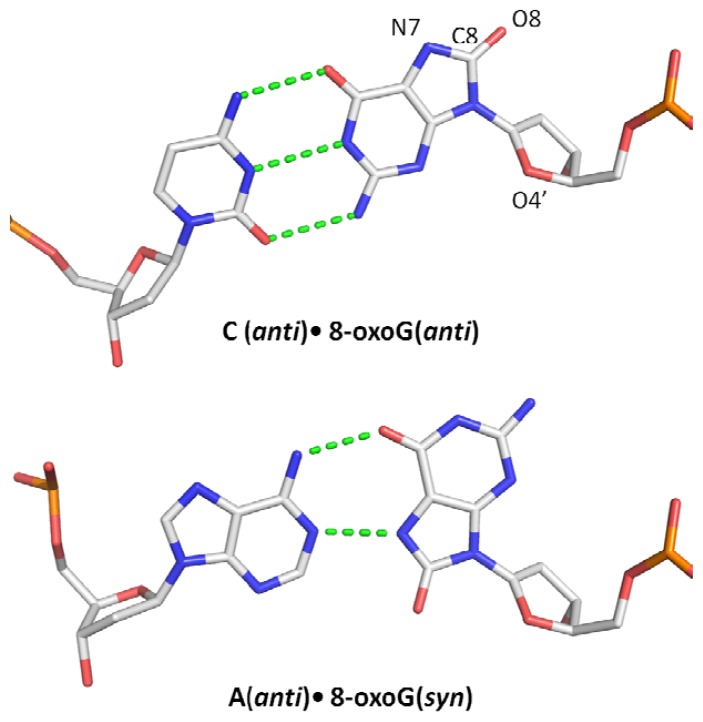
Base pairing properties of 8-oxoG. 8-oxoG forms a stable Watson-Crick base pair with cytosine. However, 8-oxoG prefers to adopt the *syn* conformation in DNA because of the steric repulsion between the 8-oxo atom and O4′ of the deoxyribose ring. In the *syn* conformation, 8-oxoG can efficiently pair with adenine, which can lead to G:C→T:A transversion after replication.

8-substituted guanine is a mixture of four tautomeric forms in solution. However, earlier spectroscopic studies revealed that the 6,8-diketo form commonly known as 7,8-dihydro-8-oxoguanine or the tautomer containing a carbonyl group on C8 and protonated N7 is the major form found under physiological conditions [10,11]. As a result, 8-oxoG has an altered arrangement of H-bond donors and acceptors making this lesion particularly mutagenic because of its miscoding properties [12]. In addition to its ability to form a Watson-Crick pairing with cytosine (8-oxoG:C), 8-oxoG has the ability to form a stable Hoogsteen pair with adenine (8-oxoG:A) (Figure 1) which can lead to a G:C→T:A transversion after replication [13–15]. Conformational studies have shown that contrary to guanine which prefers the *anti* conformation, 8-oxoG prefers to adopt the *syn* conformation in DNA because its 8-oxo group creates a steric repulsion with the deoxyribose O4′ oxygen atom in the *anti* conformation [11,16,17]. Along with its preferred *syn* conformation, the presence of a proton on the N7 atom allows 8-oxoG to mimic a thymine thereby promoting the pairing of 8-oxoG with adenine during replication [18,19].

Given the high mutagenic potential of 8-oxoG it is not surprising that living organisms have developed mechanisms to remove it from their DNA. Evolution has devised specialized enzymes that can find and remove 8-oxoG from oxidatively damaged DNA. One such enzyme family is the 8-oxoguanine DNA glycosylase (OGG), which catalyzes the first step in the base excision repair pathway (BER). Removal of 8-oxoG by OGGs is critical to prevent genomic instability and to allow the correct transmission of genes from one generation to the other. It is conceivable that the 8-oxoG removal mechanism emerged quite early in evolution, which may explain why OGGs are widespread across all three domains of life.

## 2. 8-oxoguanine DNA Glycosylase

### 2.1. OGG, Three Families, One Lesion

**Figure 2:**
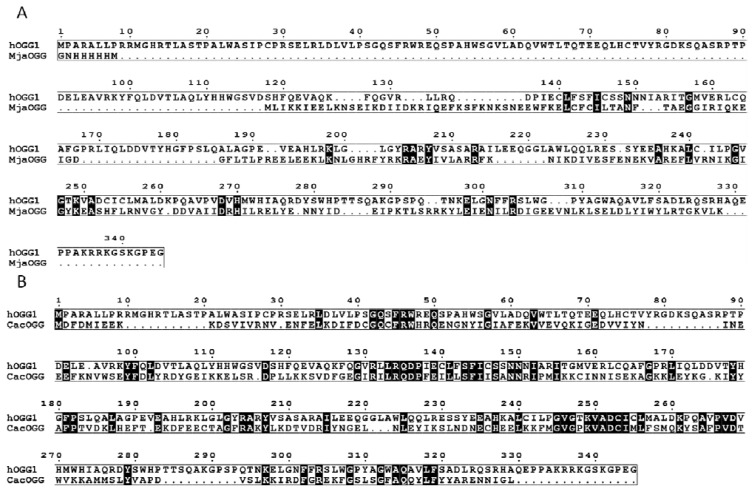
Sequence alignment of selected 8-oxoguanine DNA glycosylase (OGG) enzymes. (**A**) Structure based sequence alignment of hOGG1 (PDB ID: 1KO9 [26]) (OGG1 subfamily) and MjaOGG2 (PDB ID: 3FHF [27]) (OGG2 subfamily) illustrating the lack of *N*-terminal A-domain in OGG2; (**B**) Structure based sequence alignment of OGG1 members showing the divergence of amino acids between human and bacterial OGG1 (hOGG1, PDB ID: 1KO9 [26]; CacOGG1 PDB ID:3F0Z [25]). (hOGG1 = human OGG1 Uniprot #O15527, CacOGG1 *C. acetobutylicum* OGG1 Uniprot #Q97FM4 and MjaOGG2 = *M. janischii* OGG2 Uniprot #Q58134). Black boxed residue indicates strict conservation.

OGGs belong to the helix-hairpin-helix (HhH) superfamilly of base excision repair DNA glycosylases. HhH enzymes are widespread across all three domains of life suggesting an ancient origin [20]. It is noteworthy that OGG are not the only enzymes able to cleave 8-oxoG from DNA. Fpg which belongs to H2TH family (or the Fpg/Nei superfamily of DNA glycosylases of the BER pathway) and Nth of the HhH superfamily also excise 8-oxoG [21–23]. OGG members are divided into three subfamilies OGG1, OGG2 and AGOG, which correspond more or less to the three kingdoms of life [24]. The majority of OGG1 enzymes are found in eukaryotes whereas some OGG1 enzymes are found in a few bacterial species mostly from the Firmicutes phylum [20]. OGG2 members are found in both bacteria and archaea while AGOG are exclusively found in archaeal organisms. Despite their similar helix-hairpin-helix motif, OGG members share very little sequence homology beyond the helix-hairpin-helix core. For example, OGG2 members are completely devoid of the *N*-terminal domain of OGG1 and share only 13–19% sequence identity with OGG1 (Figure 2A). Even members of OGG1 can exhibit very low sequence homology with one another: the bacterial OGG1 from *Clostridium acetobutylicum* (CacOGG1) shares only 28% identity (Figure 2B) with its human counterpart (hOGG1) [25]. Despite these amino acid variations, OGG enzymes share analogous 3-dimensional architecture elements such as the HhH motif and are believed to possess the same catalytic mechanism based on the similarity of their active site and the conservation of their catalytic residues.

### 2.2. Overall Fold of OGG

**Figure 3:**
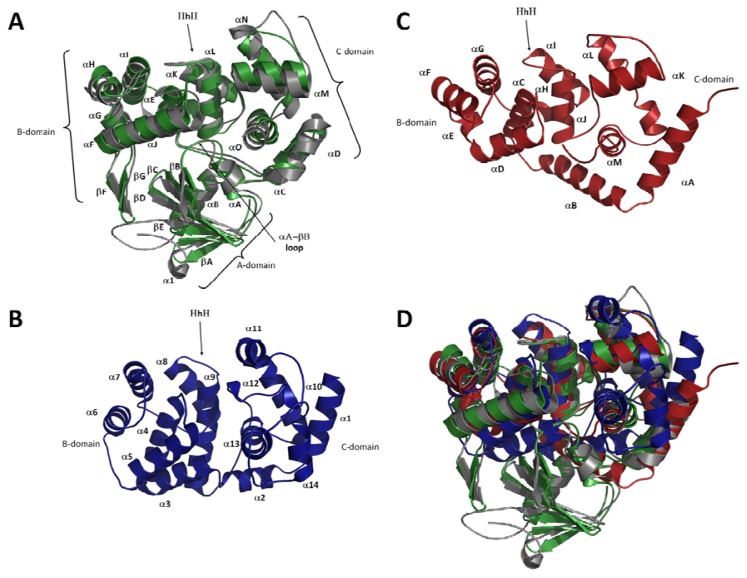
Crystallographic structures of selected OGG enzymes from the three subfamilies. (**A**) Superposition of two structures of the OGG1 clade (gray = hOGG1 PDB ID: 1KO9 [26], green = CacOGG1 PDB ID: 3F0Z [25]); (**B**) Structure of a representative member of the OGG2 subfamily (red = MjaOGG2 PDB ID:3FHF [27]); (**C**) Structure of an AGOG protein (blue = paAGOG PDB ID: 1XQO [30]); (**D**) Superposition of structures from all three OGG sub-families illustrating the similarity of the architecture of the B- and C- domains. Important structural elements are labeled.

The recent availability of OGG2 structures fills the missing gap between previously reported OGG1 and AGOG structures [26–30] and makes a comprehensive comparison possible. At first glance, the most notable difference between OGG1 and both OGG2 and AGOG is the additional *N*-terminal A-domain, formed by an antiparallel twisted β-sheet (βA to βD and αA-aB), found only in OGG1 enzymes (Figure 3A). Since OGG2 and AGOG enzymes can cleave 8-oxoG from DNA without the A-domain, the fact that this domain is conserved across OGG1 members suggests that it possesses a function yet to be identified. Interestingly, this twisted antiparallel β-sheet domain is also found in AlkA, a monofunctional glycosylase [31]. Depending on its phosphorylation state hOGG1 was observed to associate to the nuclear matrix and chromatin [32], which may be a possible function for the A-domain. This is supported by the fact that one of the two putative serine protein kinase C phosphorylation sites is located in the A-domain (Ser44PheArg). Furthermore, hOGG1 exist in two major alternatively spliced isoforms, hOGG1α and hOGG1β [33] which differ in their *C*-terminus. While hOGG1α can be found in the cytoplasm, the nucleus and the mitochondria, isoform β is exclusively expressed in the mitochondria. Interestingly, a mitochondrial translocation signal has been identified within the first 31 invariant amino acids of hOGG1 isoforms. Also, the presence of a nuclear localization signal is found only in the hOGG1α isoform which could explain its widespread expression in the cell. Deletion of the mitochondrial translocation signal in the hOGG1 A-domain seems to prevent its localization to the mitochondria [33]. Similar results have been obtained with an *N*-terminal 11 amino-acid deletion of yOGG1 [34], which supports the role of the A-domain in protein localization. It is noteworthy that bacterial OGG1 enzymes such as *Clostridium acetobutylicum* OGG [24] also harbor the OGG1 A-domain. Since bacteria are devoid of a nucleus or mitochondria is there another possible role for the OGG1 A-domain? The human Protein Reference Database [35] lists several protein-protein interactions for hOGG1 including protein kinase C, XRCC1, chromogranin B, and small nuclear ribonucleoprotein polypeptide F. It is thus possible that the A-domain may act as an anchor involved in protein interactions.

In addition to the A-domain, OGG1 members (but not OGG2 or AGOG) have a short antiparallel β-sheet formed by two β-strands (βF–βG) extruding from the B-domain. This β-sheet, first described in hOGG1 [26,29], has no known biological functions and does not interact with DNA despite its conservation. The remaining topology of the B-domain is well conserved across all three OGG subfamilies and is formed by a bundle of six α-helices and contains the HhH-GPD motif. The B-domain comprises βF–βG and αE–αJ in OGG1, αB–αJ in OGG2 and α3–α9 in AGOG (Figure 3). AGOG appears to lack helix αJ (according to OGG1 nomenclature), which connects the B-domain to the HhH motif. Even though the topology of the B-domain is conserved, the overall structures vary considerably in size. For example, hOGG1 is made of 345 amino acids whereas the bacterial CacOGG1 (also an OGG1 member) is significantly shorter with only 292 residues, although these enzymes share a very similar architecture [25]. The reduced size of CacOGG1 is reflected in shortened α-helices by an average of one helix turn and shorter connecting loops (Figure 3A).

Among all the domains found in OGG, the structure of the *C*-terminal domain (C-domain) is the most variable one. The C-domain encompasses αC, αD and αK-αO in OGG1, αA and αK-αM in OGG2 and α1, α2 and α10–α13 in AGOG (Figure 3). It is not surprising since this domain not only includes residues of the *C*-terminal part of OGG1, OGG2 and AGOG, but also can comprise residues from their *N*-terminal sequence. In both eukaryotes and bacterial OGG1, the C-domain consists of five α-helices, two of which (αC and αD) originate in the *N*-terminal sequence. In OGG2 the C-domain is formed by four α-helices and comprises both the *N*-terminal and the *C*-terminal amino acids. Helix αD of OGG1 appears to be structurally conserved with the OGG2 helix αA. The AGOG C-domain is quite different from that seen in OGG1 and OGG2: only three α-helices (α10, α12 and α13) of the AGOG C-domain are topologically conserved compared to OGG1 [30] and it has four additional helices (α1, α2, α11 and α14) with no structural counterparts in either OGG1 or OGG2. It is possible that the *C*-terminal residues (~20 a.a.) not observed in the hOGG1 models may adopt a secondary structure similar to helix α14 and its *C*-terminal loop in AGOG.

### 2.3. The Helix-Hairpin Helix (HhH) Motif

**Figure 4:**
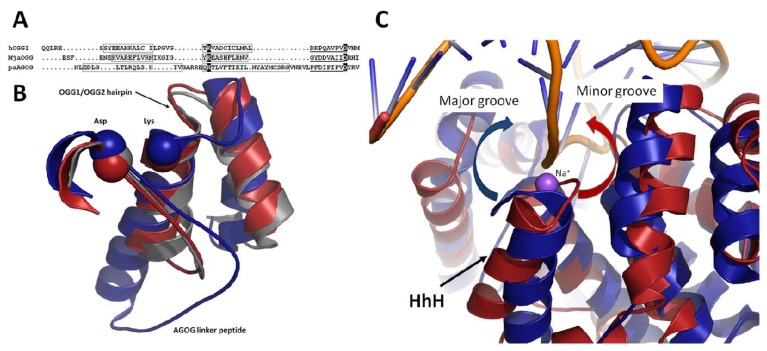
Close-up view of OGG helix-hairpin-helix-GPD motif. (**A**) Structure based alignment of the HhH-GPD motif from representative members of all three OGG subfamilies. Boxed residues indicate the helices of the motif while a black overlay indicates the strictly conserved aspartate and lysine catalytic residues. Underlined residues indicate the GPD regions of the motif; (**B**) Structure superposition of the HhH-GPD motif from hOGG1 (gray), MjaOGG2 (red) and paAGOG (blue) showing the unusual position of the hairpin in AGOG. Furthermore, the elongated helix of the HhH-GPD motif of AGOG is accompanied by a longer peptide linker. The catalytic residues of all OGG members superimpose perfectly and are depicted by a sphere around their Cα; (**C**) Putative interaction of paAGOG (blue) HhH with DNA showing that the hairpin may bind DNA toward the major groove instead of the minor groove as shown in the MjaOGG2/DNA (red) complex structure. paAGOG (PDB ID: 1XQP [30]) is superimposed onto MjaOGG2 in complex with DNA (PDB ID: 3KNT [28]). The sodium atom linking the HhH motif to a DNA phosphate group in the MjaOGG2/DNA structure is depicted as a pink sphere.

DNA-binding proteins vary considerably in the way they interact with their substrates. Many DNA-binding proteins bind DNA in a sequence-dependent manner using short specialized motifs like the helix-turn-helix or zinc fingers [36] to perform specialized cellular task such as gene regulation. However, since DNA damage occurs randomly, DNA-binding proteins have evolved motifs that bind DNA in a sequence-independent manner such as the helix-hairpin-helix (HhH) [37]. It is now widely known that the HhH-GPD motif (an HhH motif followed by a Pro/Gly-rich stretch and a conserved aspartic acid) is the hallmark of the BER superfamily, which comprises the OGG and Nth glycosylases [20,38]. Contrary to particular recognition motifs that match specific DNA motifs, HhH are very well conserved among glycosylases and bind the DNA backbone rather than specific nucleotide sequences. The highly conserved hairpin segment of the HhH usually binds to two DNA phosphates and to a cation metal (usually a calcium or sodium) that bridges the hairpin to the DNA backbone (Figure4C) [39]. There are virtually no conformational changes of the main-chain atoms of the OGGs HhH motif or the metal ion upon binding to DNA. The HhH-GDP motif in OGG1 and OGG2 is very well conserved structurally whereas in AGOG the HhH motif is significantly different in size, shape, and orientation (Figure 4B). Despite these notable differences the catalytic residues (Lys140 and Asp172) of AGOG’s HhH-GPD motif superimpose perfectly with their OGG1/OGG2 counterparts (Figure 4B). The most striking difference between AGOG and OGG1/OGG2 HhH-GDP motif is the position of the hairpin itself. While in OGG1/OGG2 the hairpin points towards the B-domain, AGOG’s hairpin points towards the C-domain, in the completely opposite direction (Figure 4B,C). AGOG’s hairpin is well stabilized in that conformation by hydrogen bonds and non-polar interactions. The non-canonical hairpin conformation is thus predicted to be unaffected by DNA binding [30]. In the absence of an AGOG structure in complex with DNA, we speculate that AGOG DNA binding differs from what was observed in the OGG1/OGG2 complexes with DNA. We predict that the hairpin of AGOG may bind DNA toward the major groove in contrast to the hairpin in OGG1/OGG2, which binds toward the minor groove (Figure 4C). A superposition of the unliganded AGOG structure (PDB ID: 1XQO) [30] with MjOGG2 or hOGG1 DNA complex structures (PDB ID: 3FHF and 1EBM, respectively) [27,29] shows that AGOG’s hairpin may bind DNA via the side chain of Arg108 and Gln139, rather than their main-chain amide group [30] as found in OGG1 and OGG2. Furthermore, the second helix of the AGOG HhH-GPD motif is about twice the size of that in OGG1/OGG2. AGOG thus requires a longer linker peptide to connect the second helix of the HhH motif to the helix harboring the catalytic aspartate to maintain the geometry of the active site. It is noteworthy that the GPD region following the linker peptide of AGOG HhH superimposes well with that of OGG1 and OGG2.

### 2.4. 8-oxoG Recognition

**Figure 5:**
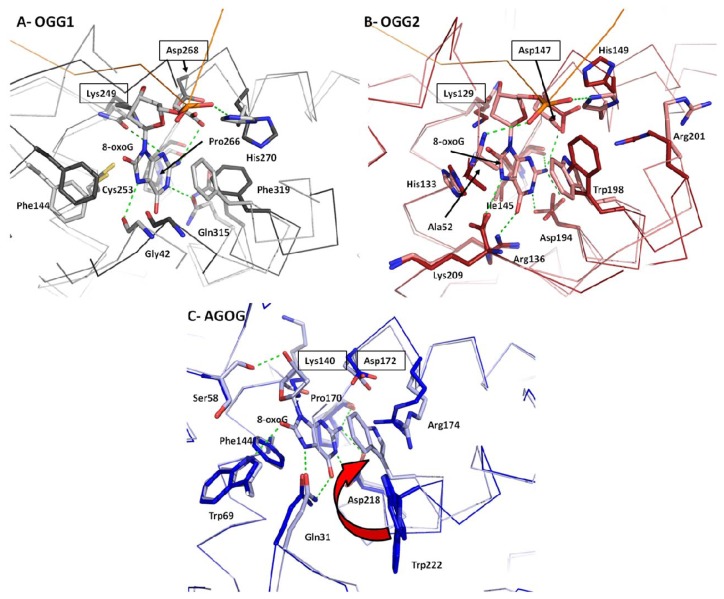
Close-up view of the 8-oxoG binding pocket. The figure shows the movement displayed by amino acids upon binding of the 8-oxoG substrate. (**A**) Apo hOGG1 is depicted in dark gray and hOGG1 in complex with DNA is depicted in pale gray (PDB ID: 1KO9 and 1EBM respectively [29,30]); (**B**) Apo MjaOGG2 and MjaOGG2 in complex with DNA containing 8-oxoG are depicted in red and pink, respectively (PDB ID: 3FHF and 3KNT [27,28]); (**C**) Apo paAGOG and paAGOG in complex with the 8-oxodG nucleoside are colored in dark and light blue, respectively (PDB ID: 1XQO and 1XQP [30]). The red curved arrow indicates the large conformational change affecting paAGOG-Trp222 upon 8-oxodG binding. Boxed labeled residues indicate the position of the conserved catalytic amino acids. H-bonds involved in 8-oxoG binding are depicted by green dashed lines. Water molecules were omitted for clarity.

Along with the OGG catalytic mechanism (see below), topics related to 8-oxoG recognition, binding and selectivity *vs*. guanine are still a matter of debate despite many recent advances. However, some consensus can be found. At first glance, the difference between 8-oxoG and guanine appears to be subtle. Oxidation of the C8 atom to form the 8-oxo group occurs with the concomitant addition of a hydrogen to the N7 atom. The first structure of an OGG bound to DNA containing 8-oxoG, hOGG1 (PDB ID:1EBM) [29] revealed that the 8-oxo moiety was devoid of any contact with the protein. The protonated N7, on the other hand, was H-bonded to the carbonyl of a strictly conserved glycine residue (Gly42) from the αA-αB loop of the A-domain (Figure 5A). The interaction between that glycine and the N7-H atom of 8-oxoG was further confirmed by the crystal structure of CacOGG1 in complex with DNA containing 8-oxoG or free nucleoside [25,39]. Attempts to abolish the Gly-N7-H bond by mutating hOGG1-Gly42 to an alanine resulted in a mitigated role of this “8-oxoG sensor” because of the steric hindrance created by the alanine methyl group [40]. Since Gly42 is strictly conserved across all OGG1 enzymes and guanine would be unable to participate in such H-bond, the role of the glycine residue in 8-oxoG specificity was easily established. However, the fact that OGG2 enzymes are devoid of the OGG1 A-domain and thus its conserved glycine raised the question of how OGG2 discriminates between 8-oxoG and guanine. Crystal structures of *Methanococcus janischii* OGG (MjaOGG2), an OGG2 enzyme, in both apo form and in complex with DNA containing 8-oxoG (PDB ID: 3FHF and 3KNT respectively [27,28]) revealed that the carboxyl group of the strictly conserved *C*-terminal lysine in OGG2 structurally overlaps with Gly42 in hOGG1 [27,28] (Figure 5B). The position of this *C*-terminal lysine is conserved in two other OGG2 enzymes (*Sulfolobus solfataricus* OGG (SSoOGG2) PDB ID: 3FHG and *Thermotoga maritima* OGG (TmaOGG2) PDB ID: 3N0U [27]). The conservation of the lysine residue is explained by the tight and highly specific binding of its side chain amino group by numerous H-bonds from the protein core. Fortunately, the location of the “8-oxoG sensor residue” at the very *C*-terminus of the enzyme provided the opportunity to generate truncated constructs to further study the role of this H-bond acceptor, something that would be impossible to do in OGG1 (PDB ID:2NOE [41]). A *C*-terminus truncated OGG2 construct failed to cleave 8-oxoG from substrate DNA while retaining its AP-lyase activity *in vitro*, strongly suggesting a crucial role for the N7-H bond in 8-oxoG/G distinction [27]. Even though the role of the OGG2 *C*-terminal lysine seems well established in distinguishing between G and 8-oxoG, Gly42 in hOGG1 may not be the only factor OGG1 uses to selectively bind its substrate because hOGG1 cleaves substrates devoid of N7-H, such as Me-Fapy-G or 7-methyl-8oxoG [42–45].

In contrast to OGG1/OGG2, AGOG appears to be the only OGG subfamily to exploit the 8-oxo group in addition to the N7-H bond to bind 8-oxoG. As seen in the AGOG structure in complex with 8-oxodG (PDB ID: 1XQP [30]), the 8-oxo group is stabilized by a H-bond formed with Trp69 (conserved in AGOGs) while the N7-H is bonded to Gln31 (also conserved) (Figure 5C). However, a mutagenesis study of AGOG showed that the interaction between the 8-oxo atom and Trp69 is not essential for recognition of 8-oxoG while N7-H bonding to Gln31 is needed. Furthermore and unlike other OGG, AGOG seems to distinguish 8-oxoG via stacking interactions involving Trp222 [30]. This residue undergoes a very large movement to stack against the 8-oxodG rings and appears to be very important for 8-oxoG recognition [30,46].

The addition of two extra atoms on 8-oxoG compared to the normal base guanine has a significant effect on the electrostatic charge distribution of the modified base. In fact, the electrostatic dipole on C8 and N7 atoms is completely inverted when guanine is oxidized to 8-oxoG. Electrostatic surface calculation of the hOGG1 8-oxoG binding pocket indicates the presence of a dipole formed by Lys249(NH_3_^+^) and Cys253(S^−^) that perfectly matches the charges of the 8-oxoG dipole [25,40] favoring 8-oxoG over guanine. Free energy calculations of 8-oxoG *vs.* guanine binding to the active site showed a 10^5^-fold preference for 8-oxoG over guanine. This Lys-Cys dipole charge difference could represent an additional feature OGG enzymes use to discriminate between 8-oxoG/G. However, a recent study of hOGG1 separation-of-function mutants revealed that the “dipole effect” does not affect cleavage of 8-oxoG [47]. A hOGG1 inverted-dipole mutant was created by switching the Lys249 and Cys253 positions, in addition to a dipole-deficient mutant (Cys253Ala). The results [47] showed no significant change in 8-oxoG processing compared to wild-type hOGG1. It thus appears that the observed dipole is not mandatory for 8-oxoG discrimination or catalysis. This observation seems to be corroborated by the structure of hOGG1 in complex with guanine (PDB ID: 3IH7 [48]) in which the guanine base occupies the exact same position as observed for 8-oxoG in the hOGG1-8oxoG DNA complex (PDB ID: 1EBM [29]). The possible contribution of the “dipole effect” on 8-oxoG binding is unlikely to occur in OGG2 or AGOG in which the residue corresponding to Cys235 in hOGG1 is a histidine in OGG2 or a phenylalanine in AGOG. In summary, the mechanism of 8-oxoG/G distinction may be multi-factorial in OGG1 but it appears to be more straightforward in OGG2 or AGOG.

### 2.5. Conformational Change upon 8-oxoG Binding

Binding of a large molecule substrate such as DNA is not without consequence on the protein conformation. In general, OGG domains tend to slightly shift toward the DNA molecule that lies in the deep middle groove of the enzyme causing a closure of the domains. This is especially true with the C-domain that undergoes several conformational changes. However, most of these changes are found in or near the active site with consequential adaptation of surrounding structural elements. In fact, the presence of an 8-oxoguanine nucleoside (8-oxodG) seems to be sufficient to promote most of the observed conformational changes. For example, the complex structure of AGOG with 8-oxodG displays shifts of numerous α-helices toward the active site compared to the apo-enzyme. This was further confirmed with the crystal structures of CacOGG1 (a bacterial OGG1) in complex with 8-oxodG and with DNA [25,39]. CacOGG1 is the only OGG crystallized in apo-, 8-oxodG and DNA-bound forms (PDB ID: 3F0Z, 3F10 and 3I0W, respectively [25,39]), providing valuable information on OGG plasticity. Comparison of CacOGG1 structures reveals small conformational changes between 8-oxodG and DNA-bound forms while significant conformational deviations are observed when compared to the apo form [25,39]. In fact, the only difference between 8-oxodG and DNA-bound forms of CacOGG1 lies in a small connecting loop (αM-αN) that is shifted due to a steric hindrance caused by the DNA backbone.

While few conformation changes are observed for the protein main chain, side-chain reorganization is found in the vicinity of the 8-oxoG binding site. The most striking conformational changes involve a conserved bulky residue that undergoes a large reorganization after 8-oxoG (or 8-oxodG) binding (Figure 5). In OGG1 a conserved Phe (Phe319 in hOGG1 and Phe282 in CacOGG1) (and the whole helix αO bearing the Phe) moves from a distal position to a stacking position forming a π-like sandwich with the 8-oxoG six-membered ring and another phenylalanine. This Phe319 reorganization over 8-oxoG allows the rotation of Gln315 (Gln278 in CacOGG1), which forms a H-bond with the N1 atom of 8-oxoG. The same situation occurs in both OGG2 and AGOG with a conserved tryptophan (Trp198 in MjaOGG2 and Trp222 in paAGOG), but in contrast to the OGG1 Phe, the moving tryptophan stacks against both rings of 8-oxoG. In addition, Asp194 in OGG2 and Asp218 in AGOG, which are structurally equivalent to Gln315 in OGG1, do not move upon 8-oxoG binding. It is noteworthy that the residue interacting with the N7-H atom of 8-oxoG which is largely responsible for the 8-oxoG/G distinction in all OGG (see previous section) remains in place and does not undergo any noticeable movement upon binding the oxidized guanine.

Residues that contact 8-oxoG are not the only ones that move upon DNA binding. Several amino acids interacting with both DNA bases and DNA backbone display changes in their conformation to bind or accommodate DNA. In hOGG1 the αE-αF loop and its NNN motif display a significant reorganization after DNA binding, shifting its position by as much as 4–9 Å [26]. The hOGG1 NNN motif interacts mainly with the DNA backbone through a main-chain amide group. It is noteworthy that the *N*-terminus Asn of the NNN motif occupies the position left vacant in the DNA double helix by 8-oxoG flipping into the binding site [29]. In the bacterial CacOGG1, however, the αE-αF loop and the degenerated NN motif do not move upon DNA binding and seem to be already in place to bind the DNA backbone. In CacOGG1 as well as in OGG2, the Asn corresponding to the hOGG1 NNN motif inserts itself into the DNA duplex with minor reorganization of its side chain. Finally, one more significant structural change is observed upon DNA binding involving either a His or a Trp in either OGG1 or OGG2 and the phosphate group of the 8-oxoG nucleotide. In hOGG1, His270 gets pushed away by Phe319 flipping, which in turns allows the formation of a H-bond with the phosphate group of the 8-oxoG nucleotide. A similar situation is observed with Trp243-Phe282 in CacOGG1 and His148-Trp198 in MjaOGG2. Unfortunately, the lack of a structure of AGOG in complex with DNA prevents us from fully analyzing possible structural reorganization in this sub-family of glycosylases.

### 2.6. Catalytic Mechanism

Despite the lack of sequence homology, OGG enzymes are all described as bifunctional enzymes, which have both glycosylase and β-lyase activities *in vitro*. The AP-lyase activity was first identified in yOGG1 through the formation of a covalent bond between the enzyme and substrate DNA in the presence of sodium borohydride [38]. It is believed that all OGG enzymes share the same catalytic mechanism as that observed for hOGG1 [46,49]. Monofunctional glycosylases proceed through an oxocarbenium ion or an activated water molecule as the nucleophile in a single-step mechanism to cleave the glycosylic bond [31,37,47,50–53]. On the other hand, OGG uses a conserved aspartate as the catalytic residue and the DNA abasic site is cleaved 3′ of the lesion in a mechanism involving the formation of a Schiff base intermediate through a conserved lysine residue *in vitro* (see below) [54,55]. However, recent and extensive study of the hOGG1 catalytic mechanism suggests [47] that the bifunctional nature of OGG might be incidental and the monofunctional pathway is most probable under physiological conditions. In the previously proposed bifunctional glycosylase/lyase mechanism the catalytic aspartic residue (Asp268 in hOGG1) activates the nucleophilic lysine (Lys249 in hOGG1), which in turn is responsible for the formation of a covalent Schiff base intermediate. The Schiff base is then hydrolyzed with a water molecule generated from the β-elimination [29,54–56]. Unfortunately, the bifunctional catalytic mechanism does not explain some experimental observations. For example, it was shown by Morland *et al.* [57] that the AP-lyase activity of hOGG1 is inhibited in the presence of free 8-oxoG. In addition, the authors showed that the Schiff base formation was abrogated at physiological concentrations of magnesium. Furthermore, in the hOGG1 structure in complex with DNA containing an abasic site analog Lys249 is far away from the catalytic Asp268, questioning its putative deprotonation [26,56]. Also, it was demonstrated that AP sites are more efficiently repaired by pol β following APE1 then by hOGG1 [58]. Finally, the *N*-glycosylic bond cleavage is at least 10-time faster than the cleavage of an abasic site [59,60], an observation which is difficult to reconcile with the fact that both enzyme and substrate are already in place to allow the second step of the reaction. These observations lead to a proposal of a monofunctional catalytic mechanism in which the weak AP-lyase activity of hOGG1 appears to be fortuitous [47]. It is therefore most probable that an AP-endonuclease such as APE1 in human is responsible for the AP-lyase activity under physiological conditions [58–60]. Biochemical and structural analyses performed by Dalhus *et al.* [47] on separation-of-function hOGG1 mutants confirmed Asp268 as the catalytic residue responsible for the monofunctional removal of 8-oxoG. Their data also suggest that Lys249 is in fact involved in the recognition and final alignment of 8-oxoG in the catalytic site by binding the 8-oxo atom prior to the base hydrolysis, though this remains to be confirmed. They also suggest that the weak lyase activity is merely the consequence of the proximity of the ɛ-amino group of Lys249 to the C1′ deoxyribose atom rather than an essential function of hOGG1. A recent finding described in Crenshaw *et al.* [48], in which a guanine is bound in the hOGG1 active site without being cleaved by the enzyme, tends to provide additional evidence of the role of Lys249 in the correct positioning or final alignment of 8-oxoG in a catalytically favorable position.

### 2.7. Estranged Base Binding Site

**Figure 6:**
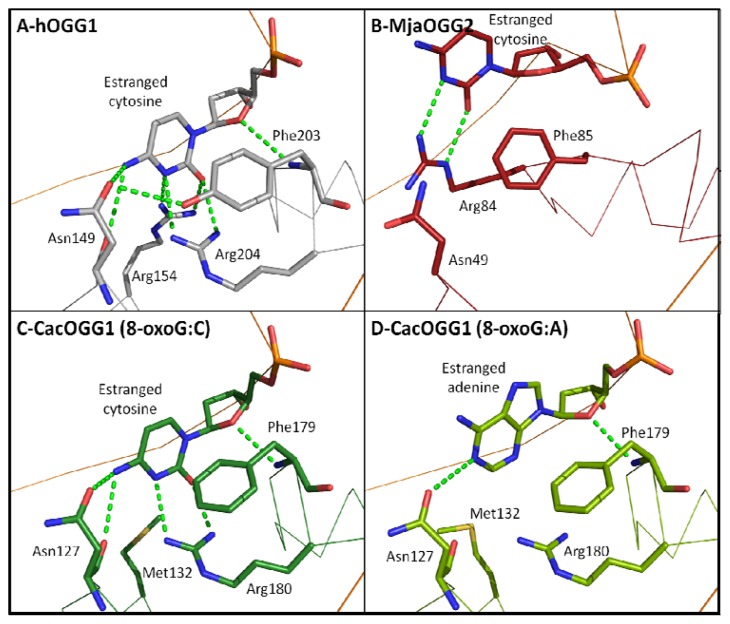
Close-up view of the interactions of OGG with the estranged base. (**A**) Interactions between the estranged cytosine and hOGG1 (PDB ID: 1EBM [29]). The cytosine is well stabilized by an extensive network of hydrophilic interactions involving two arginines (Arg154 and 204) and one asparagine (Asn149). Interaction with Phe203 locks Asn149 in a position favorable to interact with the N4 amine of cytosine; (**B**) Interactions between the estranged cytosine and MjaOGG2, an OGG2 member (PDB ID: 3KNT [28]). Only Arg84 interacts with the cytosine. Arg84 is the structural homologue of Arg204 in hOGG1. This weak stabilization of the orphaned base allows the binding of any nucleotide at this position; (**C**) and (**D**) Hydrophilic interactions between bacterial OGG1 CacOGG1 and the orphaned base in the 8-oxoG:C and the 8-oxoG:A complexes, respectively (PDB ID: 3I0W and 3I0X [39]).

In contrast to 8-oxoG recognition and the OGG catalytic mechanism, the binding of the base opposite the lesion is well understood. While eukaryotic OGG1 cleaves 8-oxoG only when paired with cytosine, bacterial OGG1, OGG2, and AGOG are less picky about the base opposite the lesion (also called the orphaned or estranged base) [24,25,28,29,39]. The availability of atomic structures of hOGG1 in complex with DNA containing 8-oxoG:C and the bacterial CacOGG1 in complex with 8-oxoG:C and 8-oxoG:A as well as the OGG2 MjaOGG2 with 8-oxoG:C provides the unique opportunity to identify key residues involved in binding the estranged base. Four residues in hOGG1 were found to interact with the opposite cytosine, namely Asn104, Arg154, Tyr203, and Arg204 (Figure 6). Tyr203 is involved in a H-bond with the O4′ of the deoxyribose of the orphaned cytosine and stacks between the cytosine and the subsequent base, causing a kink in the DNA. In addition, the hydroxyl group of Tyr203 is H-bonded to the Asn104 side-chain carbonyl thereby stabilizing the latter in a position suitable to H-bond with the cytosine. However, most of the specific binding of the estranged cytosine occurs via two arginine residues (Arg154 and Arg204) that bind the base from both sides of the ring, greatly reducing its freedom of movement. The particular arrangement of these three residues (two arginines and one asparagine) creates a unique profile of H-bond acceptors and donors that can only be matched by a cytosine. Interestingly, the natural variant Arg154His in hOGG1 was identified to have relaxed selectivity for the opposite base and is associated with colorectal tumors [61]. The bacterial OGG1 CacOGG1 is much less stringent and can efficiently cleave 8-oxoG when paired to any base [24,25,39]. The reason for this decreased specificity seems to be that the hOGG1-Arg154 structural homolog is replaced by a methionine (Met132) in CacOGG1. This change allows the cytosine to be less well stabilized and permits the binding of base with a different pattern of H-bond donors/acceptors. Furthermore, the residue corresponding to Tyr203 in hOGG1 (Phe179 in CacOGG1) cannot participate in the stabilization of Asn127, which in turn creates a more flexible binding site. This is further supported by the movement observed in Asn127 in the complex of CacOGG1 with 8oxoG:A. In that structure, Asn127 is shifted backward and its side chain is flipped by 180° to accommodate the bulkier adenine (compared to cytosine) in the binding site (Figure 6D). Site-directed mutagenesis of Met132 and Phe179 in CacOGG1 to the corresponding residues in hOGG1 (Arg154 and Tyr203) yields a CacOGG1 variant protein with a drastically increased specificity for 8-oxoG:C [24]. Finally, the decreased specificity of CacOGG1 toward the estranged base may explain why CacOGG1, unlike hOGG1, is able to cleave 8-oxoG in single-stranded DNA.

Similarly to bacterial OGG1, both OGG2 and AGOG display little or no preference for the base opposite the lesion. In hOGG1, the estranged cytosine is very tightly stabilized with four residues (Asn104, Arg154, Tyr203, and Arg204). In the structure of MjaOGG2 in complex with DNA containing 8-oxoG:C only one residue (Arg84) forms a H-bond with the cytosine; even Phe85, which corresponds to Tyr203 in hOGG1 and Phe179 in CacOGG1, does not interact with the O4′ of the ribose. The paucity of binding residues creates a wide and unspecific binding pocket allowing the binding of any of the four bases. The lack of AGOG structure in complex with DNA, along with its unconventional HhH-GPD motif and its presumably different DNA binding mode, prevents us from unambiguously assigning residues that may bind the estranged base. Finally, there seems to be a correlation between the lack of opposite base specificity and a glycosylase’s ability to cleave 8-oxoG in single-stranded DNA. Indeed, in contrast to bacterial OGG1, OGG2 and AGOG, hOGG1 is unable to cleave 8-oxoG in single-stranded DNA [24].

## 3. Conclusions

The recent availability of crystal structures of all OGG subfamilies has allowed a better understanding of the repair pathway of oxidized guanine. OGG appears to share a conserved two-domain fold with each domain flanking the HhH-GPD motif, whereas OGG1 harbors an additional *N*-terminal domain of unclear function. Sharing the same active site architecture, it is not surprising that all OGG cleave 8-oxoG using the same catalytic mechanism. New evidence tends to assign a monofunctional mechanism for OGG *in vivo* rather than bifunctional. Even if the AP lyase activity was detected by trapping the Schiff base intermediate *in vitro*, the presence of a conserved lysine in close proximity to the 8-oxoG ribose appears to be fortuitous in terms of catalysis. This lysine could be important for 8-oxoG alignment to the binding site by specifically interacting with the substrate 8-oxo group, though it remains to be confirmed. Eukaryotic OGG1 appears to have a greater specificity for cutting 8-oxoG when paired to cytosine while the corresponding bacterial and archaeal enzymes can also cut 8-oxoG paired to adenine and are active in single-strand cleavage. The increased specificity of these higher organism enzymes could be an evolutionary adaptation to the multiplicity and greater selectivity of eukaryotic DNA repair systems. To further understand OGG enzymes, a structure of a complex between an AGOG and DNA will be needed to shed light on the role of its atypical HhH-GPD motif. Crystallization of an OGG in complex with single-stranded DNA will also provide valuable information on the manner in certain OGG members bind DNA and the structural mechanisms underlying such binding.
